# From registration to insight: how STRONG AYA transforms registry data to enhance decision-support tools for adolescent and young adult oncology

**DOI:** 10.1016/j.esmorw.2026.100764

**Published:** 2026-09-15

**Authors:** N.F. Hughes, J. Hogenboom, R.N. Carter, L.J. Norman, V. Gouthamchand, O.C. Lindner, E. Connearn, A. Lobo Gomes, A. Sikora-Koperska, M. Rosińska, K. Pogoda, P. Wiechno, P. Jagodzińska-Mucha, I. Ługowska, S. Hanebaum, A.L.A.J. Dekker, W.T.A. van der Graaf, O. Husson, L.Y.L. Wee, R.G. Feltbower, D.P. Stark

**Affiliations:** 1 Leeds Institute of Medical Research, School of Medicine, University of Leeds, Leeds, UK; 2 Child Health Outcomes Research at Leeds (CHORAL), School of Medicine, University of Leeds, Leeds, UK; 3 Department of Radiation Oncology, GROW Research Institute for Oncology and Reproduction, Maastricht University Medical Centre+, Maastricht, The Netherlands; 4 Maastro, Maastricht Radiation Oncology Institute, Maastricht, The Netherlands; 5 Triply B.V., Amsterdam, The Netherlands; 6 Institute of Molecular Medicine, RWTH Aachen University, Aachen, Germany; 7 Maria Skłodowska-Curie National Research Institute and Oncology, Warsaw, Poland; 8 Digital Medicine Centre, Maria Skłodowska-Curie National Research Institute of Oncology, Warsaw, Poland; 9 Department of Medical Oncology, Netherlands Cancer Institute, Amsterdam, The Netherlands; 10 Department of Public Health and Surgical Oncology, Erasmus Medical University Centre, Rotterdam, The Netherlands; 11 Department of Medical Oncology, Erasmus MC Cancer Institute, Erasmus University Medical Centre, Rotterdam, The Netherlands; 12 Department of Clinical Medicine, Center for Clinical Data Science (CLINDA), Aalborg University, Aalborg, Denmark

**Keywords:** adolescents and young adults, clinical decision support systems, federated learning, patient-reported outcome measures, population-based cancer registries

## Abstract

**Background:**

Population-based cancer registries (PBCR) are important for monitoring trends in cancer epidemiology, facilitating the implementation of effective cancer services. Adolescents and young adults (AYAs) with cancer are a group of patients with a unique set of needs. The utility of PBCR in AYAs is limited by the lack of AYA-specific data items. STRONG AYA, an international multidisciplinary consortium, is addressing this through federated learning (FL) methodology and novel data visualisation concepts. A Core Outcome Set has been developed to measure outcomes of importance through clinical data and patient-reported outcomes (PROs). We describe how data from the Yorkshire Specialist Register of Cancer in Children and Young People (YSRCCYP), a PBCR in the UK, is being used within the STRONG AYA and how the subsequent analyses can guide patient consultation through a proof of concept.

**Methods:**

Data from the YSRCCYP were imported into an open-source privacy-enhancing FL infrastructure, from which FL analyses are carried out along with data provided by other consortium members. The results are extracted into the PROMPT software (in-house software developed by the University of Leeds and Leeds Teaching Hospitals NHS Trust) and integrated into patient electronic health care records.

**Results:**

Health care professionals can view the results of individual PROs at various time points and in comparison to summary analyses carried out within the STRONG AYA infrastructure.

**Conclusion:**

We have demonstrated how a regional PBCR can contribute to a cross-European infrastructure and analyses viewed to enhance patient consultations. Such analyses have the potential to be used for research and policy-making, improving outcomes for AYAs.

## Highlights

• STRONG AYA is a European consortium for adolescent and young adult cancer data.
• AYA registry data have been incorporated and analysed within STRONG AYA.
• Using FL analysis’ results are shared, not individual AYAs’ data.
• Novel visualisations display a patient’s own results against cross-European results.
• Innovative tools can enhance patient consultations, improving care and outcomes.

## Introduction

Cancer in adolescents and young adults (AYA), aged between 15 and 39 years, accounts for ∼5% of new cancers diagnosed in Europe.^1^ Although rare, cancer remains one of the most common causes of death in this population.^2^ For AYAs diagnosed with cancer, serious illness occurs at a key developmental time of life. This means that not only are they at risk of physical health implications but also delayed or non-achievement of developmental milestones, including educational attainment, development of relationships, and personal independence. In those who survive their cancer, the illness can result in long-lasting impairment of quality of life (QoL), affecting not only AYAs but also those who care for them and society at large.

Population-based cancer registries (PBCR) record all new cases of cancer within a defined population. They are important for monitoring trends in cancer incidence, mortality, and survival over time, facilitating the setting of policy priorities and the planning of services. Although detailed research can be carried out using PBCR in common cancers, in AYA cancers, the utility is often limited by the lack of AYA-specific data items collected relating both to the distinct cancers common in this population, and the need to assess long-term impact on QoL.

In 2016, a report by the National Cancer Institute AYA Cancer Progress Review Group recognised the value of pooling cohorts, including PBCR, for research purposes in AYA care.^3^ Further support was given by the AYA Working Group of the European Society for Medical Oncology and the European Society for Paediatric Oncology (SIOP Europe), who concluded that rapid solutions to ‘speak the same language’ among AYA cancer professionals were essential to improve outcomes for AYAs. The STRONG AYA consortium^4^ was founded in 2022 based on these principles.

STRONG AYA is a cross-European consortium comprised of AYAs with lived experience, health care professionals (HCPs), researchers, and policy makers who together aim to optimise data collection, management, and application for several purposes, including clinical decision-making. Acknowledging the specific information needed to reflect the unique situation of AYAs, STRONG AYA has developed a standardised Core Outcome Set (COS)^5^ to measure clinical and QoL variables, in part through patient-reported outcomes (PROs). This COS is being collected via seven data stations across five European countries. In addition to prospective data, the STRONG AYA initiative has an inclusive perspective on re-using existing datasets: available data are harmonised to the COS, maximising data reuse^6^ and enabling additional analyses. To date, this has led to the inclusion of over 26 500 AYAs from previously conducted studies, registries, and patient records. The data are stored in decentralised data stations, local to the site of collection by each participating institution. Data are accessible for analysis using the modern privacy-enhancing technique of federated learning (FL). Here, only the results of analyses are exchanged, rather than centralising individual-level data.

Visualisation of such European-wide analyses in clinic alongside a patient’s individual data can empower patients to take an active role in their management and provide HCPs with a valuable opportunity for personalised patient care.

In this paper, we describe a proof of concept of how PBCR data from England, UK, were integrated within the STRONG AYA infrastructure to develop an innovative real-world hospital application. Specifically, we present an initial conceptualisation for visualising STRONG AYA analyses in conjunction with individual-level health characteristics, using the Hospital Anxiety and Depression Scale (HADS)^7^ as a case study.

## Materials and methods

### Data description

The Yorkshire Specialist Register of Cancer in Children and Young People (YSRCCYP)^8^ collects detailed sociodemographic and clinical information on children and young adults who have been diagnosed with cancer in the Yorkshire and Humber region since 1974. The cohort has been described previously in detail^9^ and currently holds data on over 12 500 young people. The upper age range was extended in 2023 from 29 to 39 years to align with the international definition of AYAs. Acknowledging the importance of QoL measures in AYAs, regulatory approvals were also obtained in 2023 to further enhance the scope of the YSRCCYP through linkage of PRO data. These PRO data include those prospectively collected via STRONG AYA in addition to previously collected PROs from the Leeds Teaching Hospital NHS Trust (LTHT) collected through studies led by the Patient-Centred Outcomes Research Group (PCOR) at the University of Leeds.^10^ Currently, PRO data are only available for patients treated within LTHT and not all patients held within the YSRCCYP.

### Overcoming data inclusion challenges

**Figure 1:**
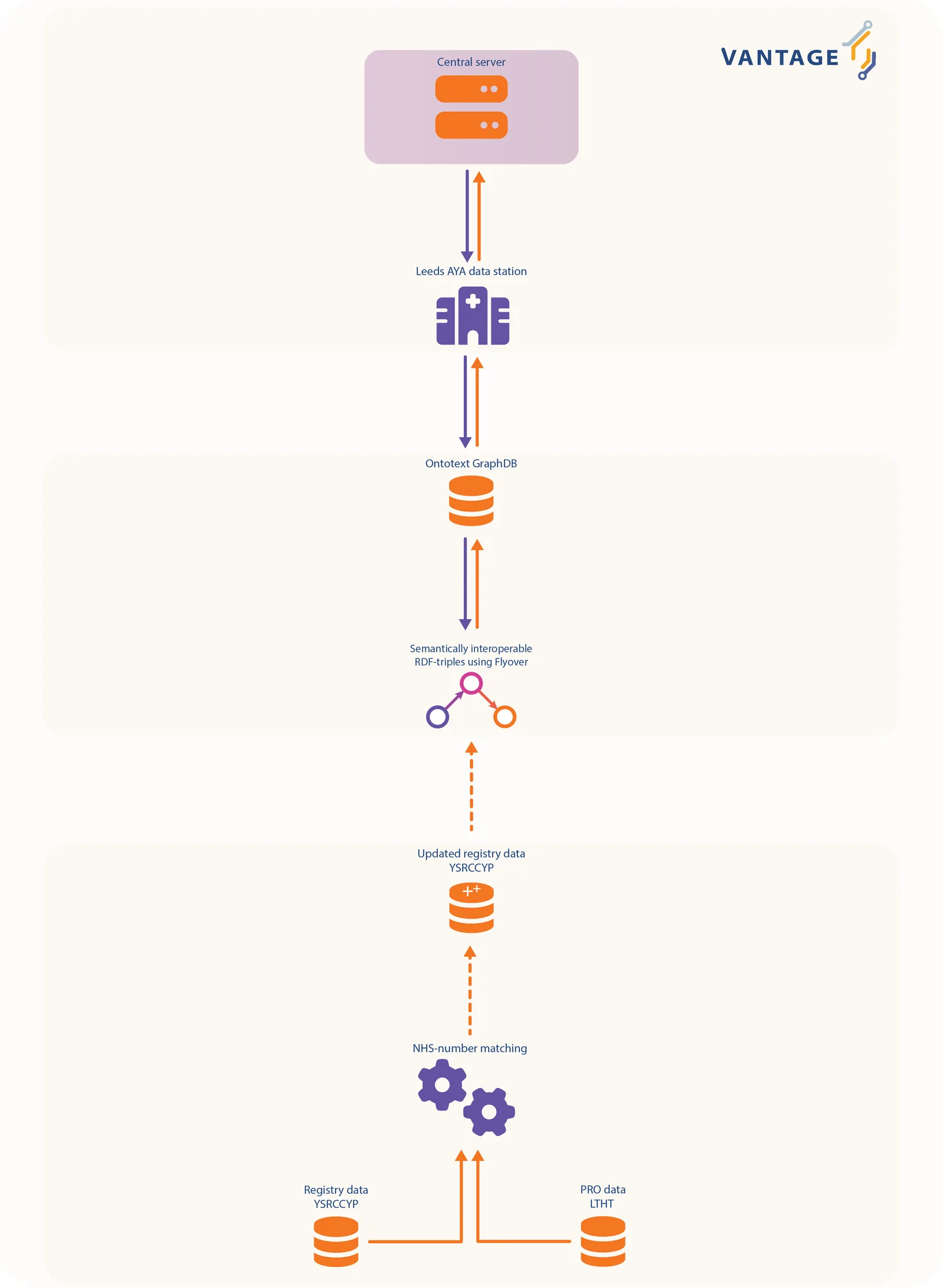
**YSRCCYP update and inclusion in the STRONG AYA federated learning ecosystem.** AYA, adolescent and young adult; LTHT, Leeds Teaching Hospital NHS Trust; RDF, resource description framework; PRO, patient-reported outcome; YSRCCYP, Yorkshire Specialist Register of Cancer in Children and Young People.

The YSRCCYP’s data format is bespoke to the region and local health care system. In international collaborations, in which harmonisation is required with other patient cohorts, this can be problematic, in particular for data related to geographically specific concepts (e.g. definition of educational levels) and further compounded in FL, in which individual-level data are not shared. In the STRONG AYA infrastructure, this challenge has been overcome by creating a shared terminology map^11^ that links data items within STRONG AYA concepts to set standards. This map is used in conjunction with the Flyover tool,^12^^,^^13^ which maps local formats on to the STRONG AYA terminology map, producing semantically interoperable resource-description-frameworks (RDF) across the FL infrastructure. A schematic overview of this process for the YSRCCYP is provided in Figure 1.

### Implementation of federated analyses in a clinical setting

LTHT, in collaboration with PCOR,^10^ co-hosts the software package PROMPT. Designed in-house at LTHT, PROMPT has been in use since 1999 to manage PROs and is integrated into the local electronic patient record system; PROMPT was developed by the University of Leeds (R.N. Carter and colleagues) for in-house use in the LTHT in Leeds. PROMPT retrieves an individual’s PROs alongside health care data and acts as a patient and HCP-facing tool to be used in consultations. Traditionally, PRO results are displayed with reference ranges derived from the clinical literature or instrument-defined ‘normal ranges’ but these are often historical and not AYA-specific. Through the STRONG AYA, we sought to provide bespoke reference ranges specific to this population.

**Figure 2:**
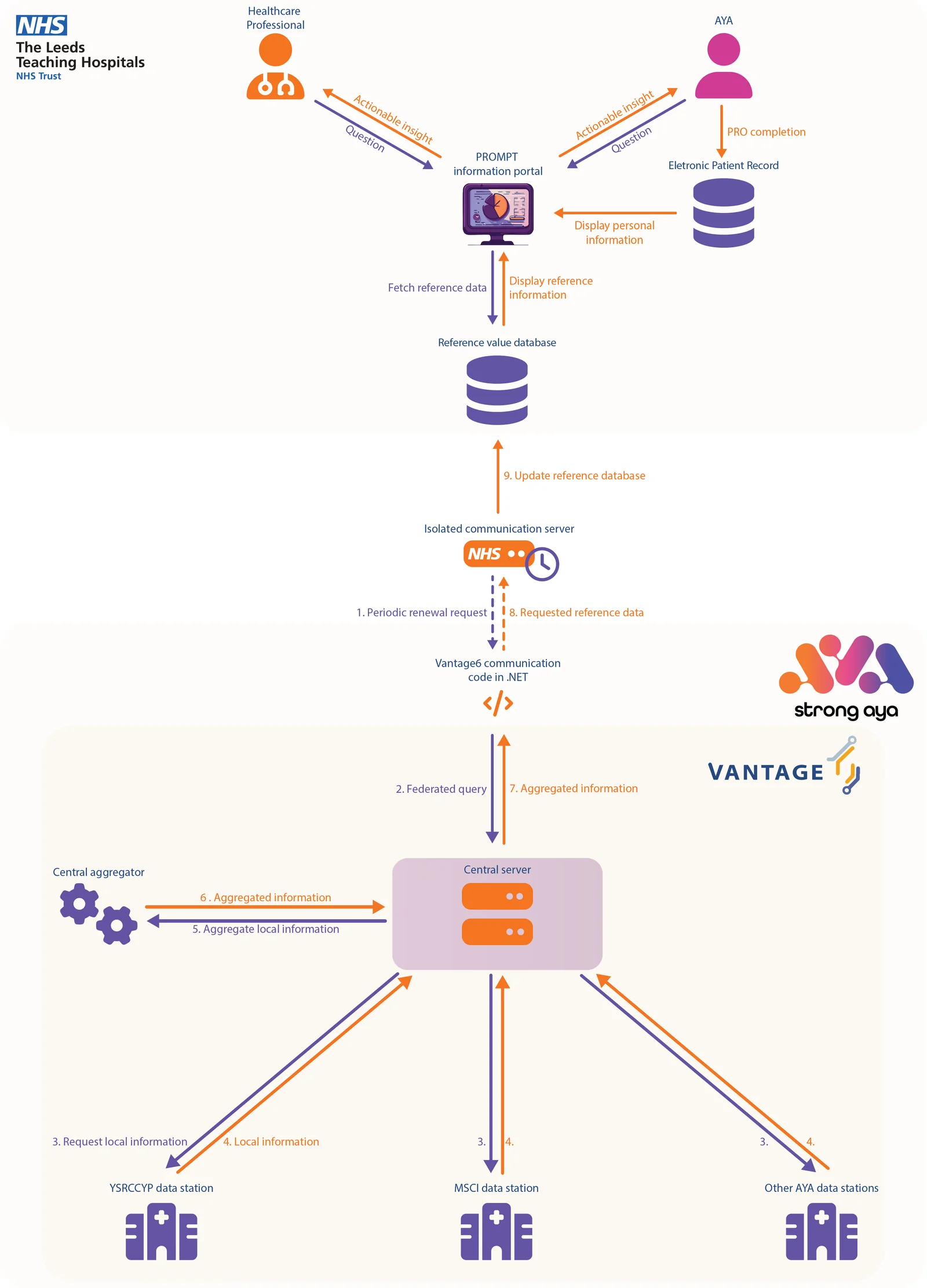
**Workflow of populating the PROMPT information portal using reference data coming from the STRONG AYA ecosystem.** AYA, adolescent and young adult; MSCI, Maria Skłodowska-Curie National Research Institute of Oncology; PRO, patient-reported outcome; YSRCCYP, Yorkshire Specialist Register of Cancer in Children and Young People.

This was achieved by implementing a bridging process^14^ between (i) the reference range within the database service hosted by LTHT and (ii) the STRONG AYA infrastructure, using Vantage 6^15^^,^^16^; an open-source FL platform. In this process, an NHS server acts as an intermediary, sending periodic queries from the LTHT-hosted database to the STRONG AYA infrastructure. The infrastructure verifies and orchestrates these requests, aggregates the local results, and returns the values to the NHS server. A schematic overview of this process and the internal PROMPT processing are illustrated in Figure 2. The implementation code bridging these two services is available on *GitHub*.

For our initial use-case, we used the STRONG AYA descriptive statistics algorithm.^17^ This algorithm computes federated descriptive statistics including sample size counts, median, and interquartile ranges and allows for detailed data stratification. The STRONG AYA descriptive statistics algorithm is open-source and available on *GitHub*.

Using this algorithm, we queried the STRONG AYA infrastructure for HADS data: ‘Total Anxiety Score’ and ‘Total Depression Score’ using their semantically interoperable codes—being *ncit:C118111* and *ncit:C118112*, respectively.^18^ The configuration file used to run this query with our implementation can be found in Supplementary Figure S1 (available at https://doi.org/10.1016/j.esmorw.2026.100764) and is available on *GitHub*.

### HCP workshop

This work was informed by an online workshop, carried out with the purpose of determining how a portal may be used in the clinical setting both from the viewpoint of HCPs and patients. Ten HCPs from a range of backgrounds, one scientific researcher, and one AYA with lived experience attended. The workshop has previously been described.^19^ A dedicated feedback session for AYAs was also carried out and attended by five participants.

## Results

### Aggregate data

The FL query revealed that 368 recordings for HADS were currently available in the STRONG AYA infrastructure (Table 1). Review of the STRONG AYA data management portal revealed that aggregates could be produced from two of the seven STRONG AYA partners, from existing observational research data, as shown in Table 1.

**Table 1:** Previously collected recordings included to date in the STRONG AYA infrastructure

Data station	Data source	Number of included individual AYA recordings	Number of HADS recordings
Netherlands Cancer Institute, Amsterdam (NL)	Registry, routine care, and research^b^	7764^a^	20
The Christie, Manchester (UK)	Routine care	6462	—
IRCCS National Cancer Institute Foundation, Milan (IT)	Registry	t.b.c^a^	—
Yorkshire Specialist Register of Cancer in Children and Young People, Leeds (UK)	Registry and research	9095	138
Centre Léon Bérard, Lyon (FR)	Routine care	3045	—
Gustave Roussy, Paris (FR)	Routine care	253^a^	—
Maria Skłodowska-Curie National Research Institute of Oncology, Warsaw (PL)	Research	210	210
Total		26 829^a^	368

Data available on the 13 February 2026.

HADS, Hospital Anxiety and Depression Scale.

a Previously collected data collating is still in progress and subject to change.

b The Netherlands Cancer Institute also contributes data from the Netherlands Cancer Registry and has national coverage.

### PROMPT integration

**Figure 3:**
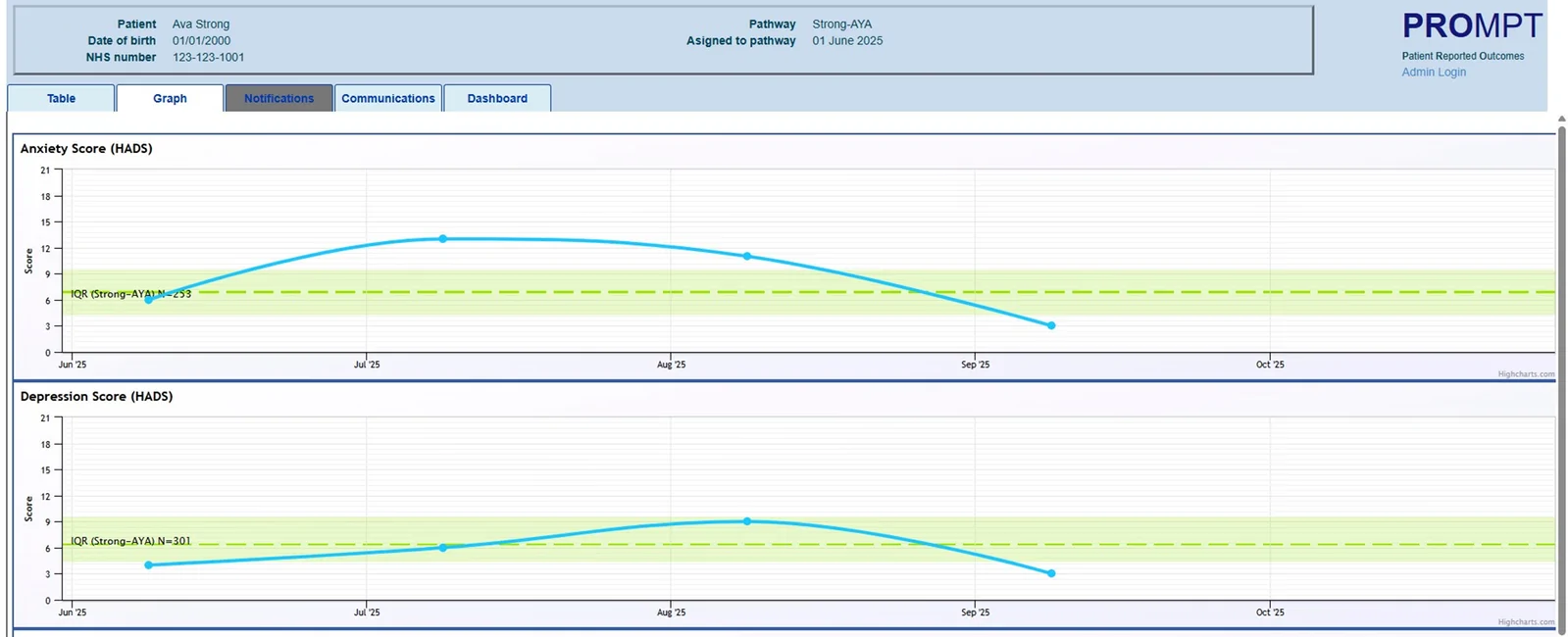
**PROMPT clinician view of patient PROs displayed as a line graph with overlaid green band showing the aggregate interquartile Q1, median (Q2), and Q3 levels determined through federated descriptive statistics returned from the STRONG AYA infrastructure.** AYA, adolescent and young adult; HADS, Hospital Anxiety and Depression Scale; IQR, interquartile range; PRO, Patient Reported Outcome.

As proof of concept, the HADS aggregated interquartile ranges have been visualised in PROMPT alongside a fictitious patient’s HADS scores. This is shown in Figure 3 and Supplementary Figures S2 and S3 (available at https://doi.org/10.1016/j.esmorw.2026.100764). Here, we illustrate HADS scores over a 3-month follow-up period, displayed in both a table and a graph. The table (Supplementary Figure S2, available at https://doi.org/10.1016/j.esmorw.2026.100764) uses a ‘traffic light’ colour-coded system: green represents scores within the STRONG AYA reference range and orange shows scores outside of this range. The line graph in Figure 3 shows longitudinal measures for the patient alongside the reference range (represented as the green overlaid band), enabling contextualisation of the patient’s scores with those of the STRONG AYA FL analysis.

Extending this use of STRONG AYA-based reference values, we were also able to display anxiety scores stratified by sex and for specific cancers for fictitious LTHT patients. This can be viewed as a distribution (histogram) chart using a dashboard view, mapping the patients’ values against the STRONG AYA reference ranges (Supplementary Figure S3, available at https://doi.org/10.1016/j.esmorw.2026.100764).

## Discussion

We describe how regional cancer registration data can be used in FL analyses within a cross-European initiative and be visualised directly within the clinical setting to aid and direct patient care.

PROs have demonstrated clinical value in facilitating the identification and monitoring of symptoms, evaluation of long-term treatment outcomes, and supporting shared decision-making.^20^, ^21^, ^22^ They have been shown to maintain patient health and QoL and to reduce burden on health care services.^23^^,^^24^ Our intention is that cross-sectional analyses presented via the HCP portal will help AYAs navigate challenges and hurdles presented to them along their treatment journey, enabling AYAs to normalise their challenges against peers of the same age with the same diagnoses across Europe. Through presentation of longitudinal analyses, we aspire to enable AYAs to conceptualise how these might change over time and identify a need for intervention where appropriate.

The success and sustainability of such an application will be dependent on positive adoption by HCPs. Previously cited barriers include scepticism, unfamiliarity, time constraints, and a preference for physiological measures.^25^ To overcome these barriers, we consulted HCPs across the consortium to ensure the product had meaningful content for users. Nonetheless, we accept that an identical portal may not be immediately applicable across all consortium members, both in terms of technological readiness and cultural barriers.

We identified that a lack of AYA-specific comparator data was a previous limitation of PROMPT. Here, we provide an approach to procure dynamic and up-to-date reference range data that can be tailored to an individual’s specific case.

### Sustainability and areas for future development

Here, we present a proof of concept for what is possible, through the STRONG AYA infrastructure and PROMPT at LTHT. The STRONG AYA infrastructure holds great potential, and across the consortium, there are several other existing HCP and patient-facing systems that can be similarly adopted to incorporate such a workflow.^26^, ^27^, ^28^, ^29^ Although here, we have focused on the visualisation of HADS reference scores, there are many other use cases for the infrastructure. Implementation of more complex models is possible and our next steps include the visualisation of more sophisticated regression models including survival analyses. Cox regression modelling and Kaplan–Meier curves will enable consortium-wide survival rates to be produced, enabling comparison of rates from individual centres or countries, and prognostic modelling.

Patient input and feedback are crucial to the acceptability and meaningful clinical utility of this HCP portal. We acknowledge that our initial online workshop included limited AYAs, and therefore, workshops are planned to obtain feedback locally at LTHT to guide ongoing development. This will ensure it is accessible and applicable to all patient groups.

The more data collected within the STRONG AYA infrastructure, the more representative and more detailed the analyses will become. Work is also underway to harmonise the HADS recordings to improve the comparability of the previously collected PROs, whereas prospective collection will increase sample size. We hope that through dissemination and ongoing collaborations, the STRONG AYA initiative will be adopted internationally by other cancer centres to improve outcomes for AYAs. We acknowledge that the collection of PRO data is a time commitment for AYAs. By demonstrating the potential benefits to patients of providing PROs and streamlining the collection process, we aspire for routine PRO collection to become an integral part of the patient care pathway.

## Conclusions

Through STRONG AYA, regional PBCR data gains added value from the incorporation of a co-developed COS for AYAs and through integration with a cross-European infrastructure. This effort enables the unique data needs of AYAs to be met and facilitates research into QoL and survivorship outcomes in a safe and secure manner. By visualising such insights within clinical settings, this approach can directly inform patient care and drive shared decision-making, thus improving AYA outcomes on an international scale.

## Supplementary data

Supplementary Fiures S1-S3

## References

[1] Trama A., Geerdes E.E., Demuru E. (2025). Survival of European children, adolescents and young adults diagnosed with haematological malignancies in the period 2000–2013: results from EUROCARE-6, a population-based study. Eur J Cancer.

[2] GBD 2019 Adolescent Young Adult Cancer Collaborators (2022). The global burden of adolescent and young adult cancer in 2019: a systematic analysis for the Global Burden of Disease Study 2019. Lancet Oncol.

[3] Smith A.W., Seibel N.L., Lewis D.R. (2016). Next steps for adolescent and young adult oncology workshop: an update on progress and recommendations for the future. Cancer.

[4] Horizon Europe (2023). The STRONG-AYA initiative: improving the future of young adults with cancer [Internet]. https://cordis.europa.eu/project/id/101057482.

[5] Janssen S.H.M., van der Graaf W.T.A., Hurley-Wallace A. (2025). Core patient-centered outcomes for adolescents and young adults with cancer: a comprehensive review of the literature from the STRONG-AYA project. Cancers (Basel).

[6] Sinaci A.A., Núñez-Benjumea F.J., Gencturk M. (2020). From raw data to FAIR data: the FAIRification workflow for health research. Methods Inf Med.

[7] Snaith R.P., Zigmond A.S. (1986). The hospital anxiety and depression scale. BMJ.

[8] Leeds Institute of Cardiovascular and Metabolic Medicine U of Leeds Yorkshire Specialist Register of Cancer in Children and Young People [Internet]. http://medhealth.leeds.ac.uk/info/545/yorkshire_specialist_cancer_register/.

[9] Cromie K.J., Crump P., Hughes N.F. (2023). Data resource profile: yorkshire specialist register of cancer in children and young people (Yorkshire Register). Int J Epidemiol.

[10] University of Leeds Patient centred outcomes research [Internet]. https://pcor.org.uk/.

[11] Hogenboom J., Gouthamchand V., Cairns C. (2026). Knowledge representation of a multicenter adolescent and young adult cancer infrastructure: development of the STRONG AYA knowledge graph. JCO Clin Cancer Inform.

[12] Gouthamchand V., Hogenboom J., Hendriks T., Wee L., van Soest J. (2026). Flyover (v3.0.2). Zenodo.

[13] Gouthamchand V., Choudhury A., Hoebers F.J.P. (2024). Making head and neck cancer clinical data findable, accessible, interoperable, reusable to support multi-institutional collaboration and federated learning. BJR Artif Intell.

[14] Hogenboom J. (2026). STRONGAYA/v6-py-client-in-dotnet: Vantage6 client in .NET (v1.0.1) . Zenodo.

[15] Smits D., Van Beusekom B., Martin F., Veen L., Geleijnse G., Moncada-Torres A. (2022). An improved infrastructure for privacy-preserving analysis of patient data. Stud Health Technol Inform.

[16] Martin F., van Beusekom B., Leurs R. (2026). vantage6 (version/4.13.6). Zenodo.

[17] Hogenboom J. (2025). STRONGAYA/v6-descriptive-statistics: Vantage6 Descriptive Statistics (v2.0.0-pre) [Internet]. Zenodo.

[18] Hogenboom J. (2025). STRONGAYA/AYA-cancer-semantic-map: STRONG AYA semantic map (v1.0.0) [Internet]. Zenodo.

[19] Hogenboom J., Košir U., Deželak T. (2025). Development of the STRONG-AYA federated learning-based adolescent and young adult cancer information portal: a co-creation process with adolescents and young adults with lived experience of cancer (preprint). JMIR.

[20] Haverman L., Luijten M.A.J., Blackford A.L. (2024). Truth and dare: patients dare to tell the truth when using PROMs in clinical practice. Qual Life Res.

[21] Santana M.J., Haverman L., Absolom K. (2015). Training clinicians in how to use patient-reported outcome measures in routine clinical practice. Qual Life Res.

[22] Gibbons C., Porter I., Gonçalves-Bradley D.C. (2021). Routine provision of feedback from patient-reported outcome measurements to healthcare providers and patients in clinical practice. Cochrane Database Syst Rev.

[23] Takeuchi E.E., Keding A., Awad N. (2011). Impact of patient-reported outcomes in oncology: a longitudinal analysis of patient-physician communication. J Clin Oncol.

[24] Aiyegbusi O.L., Hughes S.E., Peipert J.D., Schougaard L.M.V., Wilson R., Calvert M.J. (2023). Reducing the pressures of outpatient care: the potential role of patient-reported outcomes. J R Soc Med.

[25] Fontaine G., Poitras M.E., Sasseville M. (2024). Barriers and enablers to the implementation of patient-reported outcome and experience measures (PROMs/PREMs): protocol for an umbrella review. Syst Rev.

[26] Kuijpers W., Groen W.G., Oldenburg H.S., Wouters M.W., Aaronson N.K., van Harten W.H. (2016). eHealth for breast cancer survivors: use, feasibility and impact of an interactive portal. JMIR Cancer.

[27] Kuijpers W., Groen W.G., Oldenburg H.S., Wouters M.W., Aaronson N.K., van Harten W.H. (2015). Development of MijnAVL: an interactive portal to empower breast and lung cancer survivors: an iterative, multi-stakeholder approach. JMIR Res Protoc.

[28] Nederlands Kanker Institute MijnAVL: your personal patient portal [Internet]. https://www.avl.nl/en/mijnavl/.

[29] Léon Bérard Center myCLB [Internet]. https://myclb.sante-ra.fr/Home/Login/tabid/229/Default.aspx?returnurl=%2f.

