# Supplementary material for: From registration to insight: how STRONG AYA transforms registry data to enhance decision-support tools for adolescent and young adult oncology
Source: ESMO Real World Data Digit Oncol. 2026 Sep 15;14:100764. doi: 10.1016/j.esmorw.2026.100764 (PMC13594699; doi:10.1016/j.esmorw.2026.100764)
Supplement: Supplementary file 1 [file mmc1.docx]

Supplementary figure 1. Configuration of our implementation to retrieve Hospital Anxiety and Depression Scale scores.

{
 "name": "HADS scores descriptive statistics",
 "description": "Task to score and take the descriptive statistics for HADS anxiety and depression scales.",
 "databaseLabels": "rdf_store",
 "image": "ghcr.io/strongaya/v6-descriptive-statistics@sha256:b3f86469fc339a77cbc5edac139912243db8e1051d0dfcbd508321a0100dd975",
"input": {
 "method": "central",
 "kwargs": {
 "variables_to_describe": {
 "ncit:C118111": {
 "datatype": "numerical"
 },
 "ncit:C118112": {
 "datatype": "numerical"
 }
 }
 }
 },
 "algorithm_repository": "https://github.com/STRONGAYA/v6-descriptive-statistics",
 "algorithm_wiki": https://github.com/STRONGAYA/v6-descriptive-statistics/wiki
}

Supplementary figure 2. PROMPT clinician view of a patient PROMs displayed in a table with colour thresholds set to reference interquartile ranges determined through federated descriptive statistics returned from the STRONG AYA infrastructure.

Supplementary figure 3: PROMPT clinician dashboard view showing a fictitious HADS score distribution for all female patients as typically displayed in the colorectal treatment pathway; the green background is the aggregate interquartile Q1, median (Q2), and Q3 levels determined through federated descriptive statistics returned from the STRONG AYA infrastructure.
